# Skin to Intramuscular Compartment Thigh Measurement by Ultrasound in Pediatric Population

**DOI:** 10.5811/westjem.2016.12.32279

**Published:** 2017-02-07

**Authors:** Myto Duong, Albert Botchway, Jonathan dela Cruz, Richard Austin, Kevin McDaniel, Cassie Jaeger

**Affiliations:** * Southern Illinois University School of Medicine, Department of Surgery, Division of Emergency Medicine, Springfield, Illinois; † Memorial Medical Center, Department of Emergency Medicine, Springfield, Illinois; ‡ Southern Illinois University, School of Medicine, Center for Clinical Research, Springfield, Illinois

## Abstract

**Introduction:**

Pediatric obesity threatens the efficacy of medications given intramuscularly. In anaphylactic patients, epinephrine auto-injector needle lengths are potentially too short to reach the muscle compartment in patients with elevated body habitus. The objective of the study was to determine needle-length requirements for intramuscular injections in pediatric patients.

**Methods:**

We used ultrasound to measure the distance from skin to muscle compartment of the thigh in 200 pediatric patients of various weight and body mass index who presented to the emergency department.

**Results:**

Patients with higher body mass index had an increased distance to muscle and bone. If current recommendations were followed, 5% of patients within the EpiPen adult weight category and 11% of patients within the Centers for Disease Control and Prevention weight category would have potentially used a needle inadequate in length for intramuscular injections.

**Conclusion:**

With the increase in childhood obesity, needle lengths may be too short to effectively deliver medications to the intramuscular compartment. Needle length should be evaluated to accommodate pediatric patients with increased skin to muscle distance.

## INTRODUCTION

During a severe allergic reaction, anaphylaxis, certain life-saving medications such as epinephrine have a quicker onset of action if given intramuscularly.^1^,^2^ In response to allergen exposure, mast cells and basophils release inflammatory mediators, which promote a systemic reaction with potential respiratory and cardiovascular consequences.^3^–^11^ Epinephrine, an adrenergic receptor agonist, increases vasoconstriction and peripheral vascular resistance through the alpha-1 receptor, elevates inotrophy and chronotrophy through the beta-1 receptor, and promotes bronchodilation and vasodilation via the beta-2 receptor to reduce anaphylactic symptoms and promote a homeostatic state.^12^,^13^ The National Institute of Allergy and Infectious Diseases recommends epinephrine be given intramuscularly as the first line of therapy to rapidly treat anaphylaxis. Auto-injector needles that facilitate the intramuscular injection should be adequate in length to reach between the subcutaneous adipose tissue and vastus lateralis muscle.^14^ If treatment is injected intramuscularly to the anterolateral aspect of the thigh, blood levels of epinephrine are therapeutic at eight minutes. However, if treatment is given subcutaneously, therapeutic levels of epinephrine are not reached until 22 minutes, extending exposure of potentially life-threatening symptoms and therefore increasing morbidity and mortality risk.^2^

It is well known that obesity is a growing issue in the pediatric population in the United States (U.S.), with obesity statistics more than doubling in children over the past 30 years.^15^,^16^ In 2012, 16.9% of 2–19 year olds were considered obese, with obesity defined as body mass index (BMI) at or above the 95^th^ percentile of the sex-specific BMI-for-age-growth charts.^15^,^17^ With the increase in body habitus, problems of appropriate intramuscular medication delivery via standard needle lengths to these children is a great concern. The EpiPen and EpiPen Jr cartridge-based epinephrine auto-injector devices approved in the U.S. have an activated needle length of 1.6 centimeters (cm) and 1.3cm, respectively.^18^,^19^ For children 3–18 years old, the Centers for Disease Control and Prevention (CDC) recommends using a 2.54cm needle for intramuscular injection into the vastus lateralis muscle. A 2.54–3.81cm needle is recommended for those whose weight falls between 69–118 kilograms (kg).^20^

In 2009, a study including 256 children 1–12 years of age found that BMI significantly influenced distance from skin to vastus lateralis muscle when measured by ultrasound (US). The skin to muscle distance of 12% of the children who weighed less than 30kg and 30% of children who weighed greater than 30kg exceeded auto-injector needle length. Epinephrine auto-injector needle lengths are potentially too short to reach the muscle compartment for most pediatric patients.^21^ A graphical reference of BMI versus distance to the muscle compartment that physicians can use to select appropriate needle lengths for intramuscular injections is needed. The objective of this study was to determine needle-length requirements for intramuscular injections of medication or vaccines by using US to measure the distance from skin to muscle compartment of the thigh in pediatric patients of various weight and BMI. We hypothesized that distance from skin to muscle would correlate with BMI.

## METHODS

This was a prospective study that used convenience sampling of 200 pediatric patients less than 18 years of age who presented to the emergency department (ED). The study was conducted between October 2013 and August 2014. The institutional review board at the authors’ institution approved this research project and the research was conducted according to federal research guidelines.

We excluded patients if a chronic illness was present, which could have impeded normal growth or development. Other exclusion criteria included the presence of cystic fibrosis, congenital heart disease, autoimmune disorders, failure to thrive and medical resuscitation. Pediatric patients were approached to participate in the study in the ED. If they were interested in participating the study, informed consent was obtained from the child’s guardian and assent was obtained from patients older than six years of age.

An US certified pediatric emergency physician performed the ultrasounds. Depth measurement was taken at the midpoint between the superior aspects of the anterior superior iliac spine to the superior aspect of the patella, on the lateral aspect of the right thigh. Minimal but sufficient pressure on the probe was applied to get adequate US imaging. Sagittal images were obtained for measurements from the skin to the fascial layer and mid-depth of the muscle mass. Study staff recorded the measurement. Once the US measurements were made, a still US picture was obtained. The whole measurement procedure took five minutes to perform.

**Population Health Research Capsule**

What do we already know about this issue?

Needle lengths used for pediatric intramuscular injections are potentially too short to reach the muscle compartment in patients with elevated body mass index.

What was the research question?

What needle lengths are required for successful intramuscular injection in a sample of 200 pediatric patients?

What was the major finding of the study?

A small percentage of patients fell outside EpiPen and CDC needle lengths for intramuscular injection.

How does this improve population health?

Assessing skin to intramuscular distance is needed in specific pediatric populations to determine needle length and improve intramuscular medication delivery.

Patient’s age (months), height (cm) and weight (kg) were recorded to calculate each patient’s BMI. These measurements are done routinely by triage staff while the patient is in the waiting room, but if they were not done prior to patient enrollment in the study, study investigators obtained them. Other patient information collected included gender and ethnicity, which was used to determine if other factors may be good predictors for increased depth to the muscle compartment. We executed a distribution graph for BMI versus skin to muscle and bone compartment depth, measured in centimeters. Data were collected using paper forms and then uploaded into an electronic data file using Microsoft Excel (Redmond, WA). We performed Pearson correlation coefficient and linear regression in SAS version 9.4 (Cary, NC) to determine significance.

## RESULTS

Gender distribution was proportionate, with 99/200 male subjects (49.5%) and 101/200 female subjects (50.5%). Caucasian, African-American, and Hispanic subjects represented 55%, 44.5% and 0.5% of the subject population, respectively.

**Figure 1:**
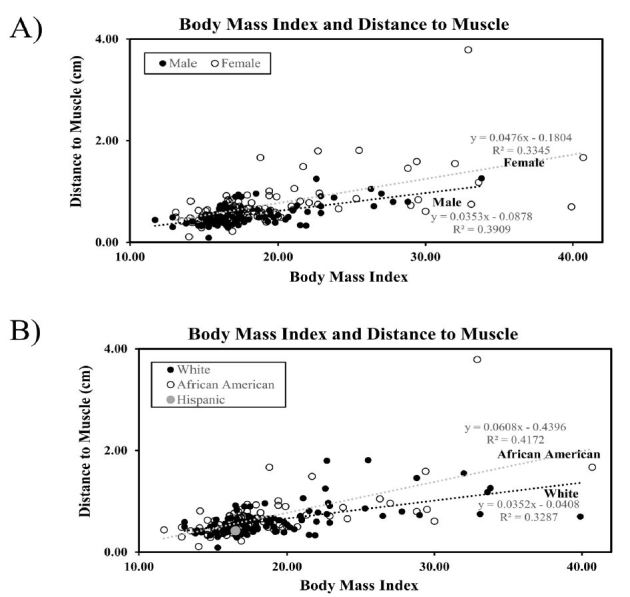
Body mass index predicts distance to muscle. Regression analysis was performed from ultrasound measurements from skin to muscle and analyzed by gender (A) and ethnicity (B). Best fit regression lines are represented for gender and ethnicity. The equation of the line for total patients was Y=0.046x–0.2142, R^2^=0.3515.

**Figure 2:**
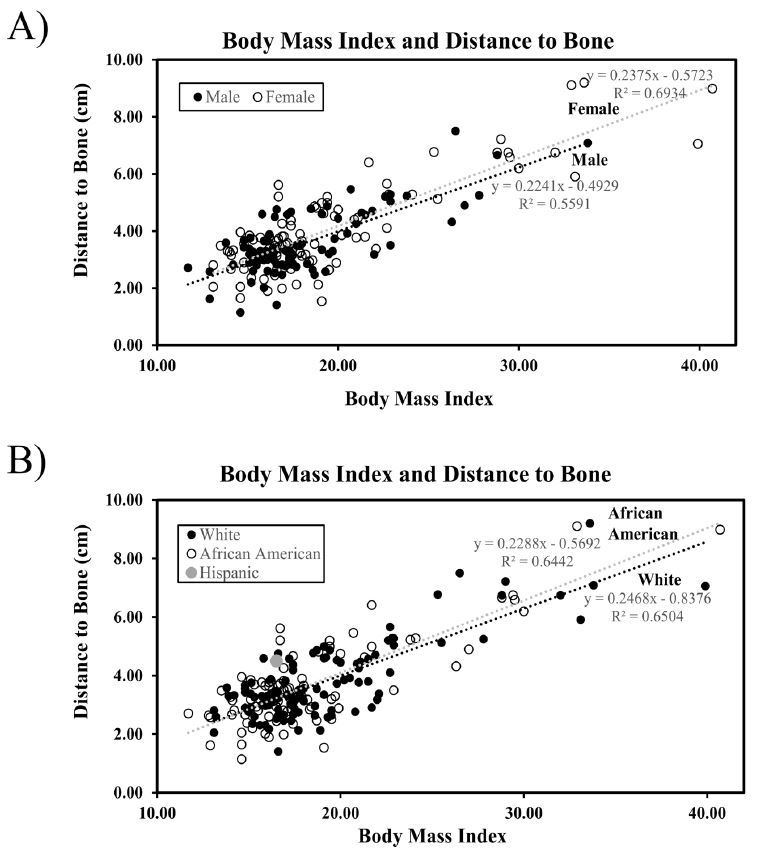
Body mass index predicts distance to bone. Regression analysis was performed from ultrasound measurements from skin to bone and analyzed by gender (A) and ethnicity (B). Best fit regression lines are represented for gender and ethnicity. The equation of the line for total patients was Y=0.2356x–0.664, R^2^=0.6429.

The mean BMI for all subjects combined averaged to 19 with standard deviation of +/− 5.3. The mean depth to muscle was 0.72cm. Regression analysis determined that BMI significantly predicted the distance to muscle and that subjects with higher BMI tended to have a greater distance to muscle, with an R^2^ value, which indicates how well the linear model fits the data, of 0.3515 and a p-value of <0.001 or Pearson correlation coefficient of 0.6 (Figure 1). Additionally, regression analysis determined that BMI significantly predicted the distance to bone and that subjects with higher BMI tended to have greater distance to bone, with an R^2^ value of 0.6429 and a p-value of <0.001 or a Pearson correlation coefficient of 0.8. The mean depth to bone was 3.84cm (Figure 2). When analyzed by gender and ethnicity, female and African-American patients had higher trends in BMI and distance to muscle and distance to bone compared to white males. The Hispanic population included less than five patients (Figures 1–2).

**Figure 3:**
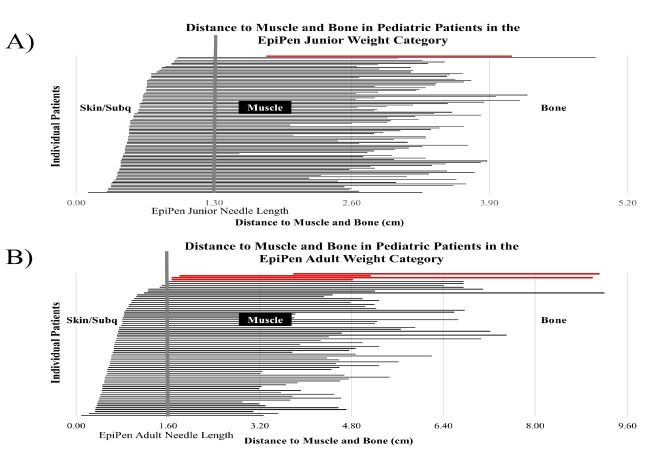
Distance to muscle and distance to bone of pediatric patients in the EpiPen Jr (A) and EpiPen adult (B) weight categories. Left end of horizontal bars represent the beginning of the vastus lateralis muscle. Right end of horizontal bars represent beginning of greater tuberosity of femur. Vertical bar represents 1.3cm (panel A) and 1.6cm (panel B) needle distance of EpiPen Jr and EpiPen adult auto-injectors, respectively. Horizontal red bars represent patients with distance from skin to muscle larger than the recommended needle length.

**Figure 4:**
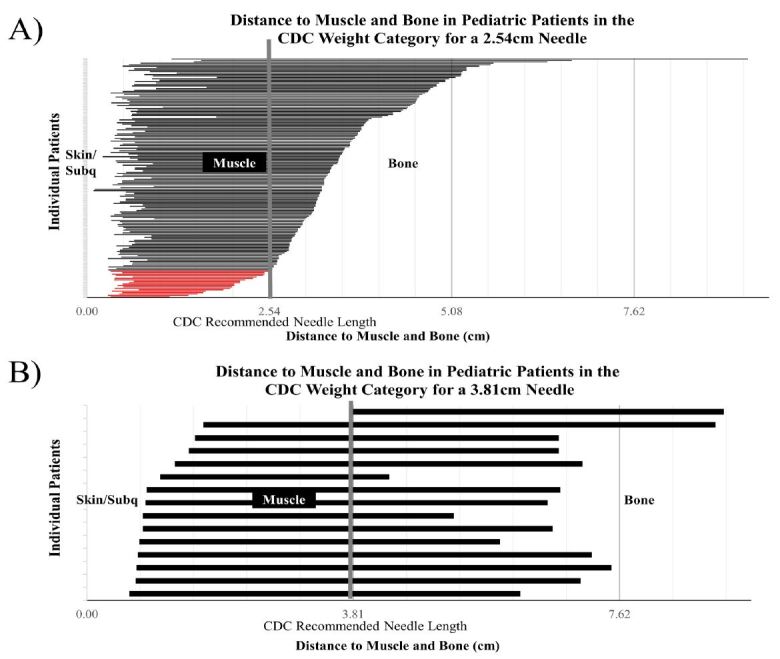
Distance to muscle and distance to bone of pediatric patients in the 2.54cm CDC needle-length range (A) and 3.81cm CDC needle-length range (B) weight categories. Left end of horizontal bars represent the beginning of the vastus lateralis muscle. Right end of horizontal bars represent beginning of greater tuberosity of femur. Vertical bar represents 2.54cm (panel A) and 3.81cm (panel B) CDC-recommended needle length. Horizontal red bars represent patients with distance from skin to muscle outside the recommended needle length. *CDC*, Centers for Disease Control and Prevention.

The relationship between distance to muscle and distance to bone was compared to EpiPen and CDC-recommended needle lengths to determine how many patients could have potentially received an inadequate intramuscular injection. Out of 110 patients who fell within the 7.5kg–25kg EpiPen Jr weight range, 0.9% (1/110) had a distance to muscle and distance to bone that was not in range with the recommended 1.3cm needle length (Figure 3A). Out of the 77 patients who fell within the ≥25kg EpiPen adult weight range, 5% (4/77) had a distance to muscle and distance to bone that was not in range with the recommended 1.6cm needle length (Figure 3B). Out of the 169 patients who fell within the CDC-recommended needle-length weight category of <69kg, 11% (19/169) had a distance to muscle and distance to bone that was not in range with the recommended 2.54cm needle length (Figure 4A). None of the 15 patients who fell within the CDC-recommended needle-length weight category of >69kg had a distance to muscle and distance to bone that was out of needle-length range (Figure 4B).

## DISCUSSION

Intramuscular injection of epinephrine during anaphylaxis allows medication to rapidly alleviate anaphylactic symptoms such as hypotension, bronchial airway constriction, and decreased cardiac output.^1^,^2^,^12^,^13^,^22^ Auto-injector needles should be adequate in length to reach between the subcutaneous adipose tissue and vastus lateralis muscle for blood levels of epinephrine to reach therapeutic levels approximately eight minutes after injection.^14^,^2^ Obesity continues to burden the pediatric population, potentially preventing appropriate intramuscular medication delivery when standard auto-injector needle lengths are used.^3^,^15^,^16^

In the current study, US measurements were performed to determine distance from skin to muscle and skin to bone in 200 subjects ranging from 0.13 to 17 years in age. BMI was determined from subject’s age (months), height (cm) and weight (kg). Subjects with higher BMI tended to have greater distance to muscle and bone. When distance to muscle and distance to bone was compared to weight-dependent needle recommendations, 5% of patients within the EpiPen adult weight category and 11% of patients within the CDC weight category could have potentially used a needle inadequate in length (Figures 3–4).

Variability of distance between skin and muscle exists within the literature. In 2008, a study measured thickness of subcutaneous fat tissue and muscle in 100 children aged two months to six years using magnetic resonance imaging (MRI) and computed tomography (CT). Average depth from skin to muscle was ~1.2cm. However, neither MRI nor CT apply pressure to the skin during measurement, which may not accurately represent auto-injector instructions for EpiPen use. Additionally, the study population’s weight and age distribution may not have represented the general population.^23^ In the current study, US measurements were obtained while the child was in a supine position with legs in extension. Since procedural EpiPen instructions recommend the user lie down with their legs slightly elevated, protocols were similar but not exact, which may have influenced needle-length estimations.^18^

In 2009, Stecher et al. used US to measure the depth from the skin to vastus lateralis muscle in 256 subjects between 1–12 years of age and reported that BMI and age were good predictors for increased depth to muscle compartment.^21^ Additionally, when depth from skin to muscle was measured in 120 adults 18–55 years old, 31% were at risk for potential undersized auto-injector needle length. Potential inadequate needle length correlated with higher BMI.^24^

It is important to note that many other medications administered in the pediatric healthcare setting require intramuscular injection to be effective. Vaccines in the outpatient setting and procedural sedation agents particularly in the ED setting are two categories of medications in which intramuscular administration is of utmost importance. In 2002, Cook et al. used US to determine needle length for intramuscular vaccinations in two-, four-, six-, and 18-month-old patients. Although needle length aligned with the World Health Organization and the National Health and Medical Research Council, it was dependent on the injecting angle technique (90 degrees versus 45 degrees).^25^ In 1997, Groswasser et al. used a high-frequency, real-time ultrasonograph to measure subcutaneous tissue and muscular layer thickness in children at the ages when common vaccinations are given. The authors reported needle length could be determined by ultrasonographic measurements and that successful injection depended on injection technique.^26^ Ipp et al. compared adverse reactions in children vaccinated with intramuscular needle lengths of either 1.6cm or 2.5cm. Redness and swelling was more common in children vaccinated with the 1.6cm needle.^27^ Additionally, in 2006 a randomized controlled trial examined reactions in infants vaccinated using either 1.6cm or 2.5cm length needles. Infants vaccinated with the longer needle had significantly less severe local reactions.^28^ Data from this and other studies suggest there are specific pediatric populations where assessing for skin to intramuscular distance needs to be performed to identify adequate needle lengths for administration.^21^ Bedside ultrasonography has a role in assessing for this distance until more data are available to create more generalizable regression models for needle-length requirements.

Clinicians and auto-injector manufactures should continue to evaluate contributing factors such as age, demographics, and BMI to work toward use of the safest and most effective needle length. Currently, nurses at our institution select intramuscular needle length based on recommendations and body habitus. The authors advocate the use of US, if cost and urgency of the procedure permits, and development of a pediatric guide that determines appropriate needle-length sizes for BMI. Specific needle-length guidelines have the potential to improve intramuscular injections not only for auto-injectors but for other pediatric vaccinations and emergency procedures requiring effective intramuscular medication delivery.

## LIMITATIONS

The average BMI for all subjects combined was 19 with standard deviation of +/− 5.3, suggesting that the majority of subjects fell into the normal or healthy weight category and the population may not have been representative of obesity prevalence (Figure 1–2).^17^ Future studies with increased sample size would likely increase the number of subjects with a BMI that falls into the overweight or obese category and increase R^2^ values to obtain a closer linear model fit. Although there was a linear correlation between skin to muscle and bone depth with BMI, with more data a non-linear curve may be more apparent.

A limitation of the current study was that intramuscular injections were not performed to record needle length used and success of the injection. Additionally, convenience sampling may not accurately represent the whole population. However, linear regression analysis determined that BMI significantly correlated with US measurement of depth of skin to muscle and skin to bone. Generation of a graph with representative demographics would be useful in determining appropriate size needles required for patients of variable BMI to ensure intramuscular administration of medications or vaccines.

## CONCLUSION

Distance from skin to muscle compartment of the thigh was measured by ultrasound in 200 pediatric patients of various weight and BMI who presented to the ED. Linear regression analysis determined that BMI significantly correlated with ultrasound measurement of depth of skin to muscle and skin to bone. When distance to muscle and distance to bone was compared to weight-dependent needle recommendations, 5% of patients within the EpiPen adult weight category and 11% of patients within the CDC weight category could have potentially used a needle inadequate in length.
